# Results of a 14-Week, Multicenter, Prospective, Randomized, Open-Label, Noninferiority Clinical Trial Comparing the Antihypertensive Effect and Edema Incidence of Lacidipine and Amlodipine in Older Korean Patients with Mild-to-Moderate Hypertension^☆^

**DOI:** 10.1016/j.curtheres.2013.02.001

**Published:** 2013-06

**Authors:** Jin Oh Na, Hong Seog Seo, Cheol Ung Choi, Hong Euy Lim, Jin Won Kim, Eung Ju Kim, Seong Woo Han, Seung-Woon Rha, Chang Gyu Park, Dong Joo Oh, ELDER investigators

**Affiliations:** Cardiovascular Center, Korea University Guro Hospital, Korea University College of Medicine, Seoul, Korea

**Keywords:** amlodipine, blood pressure, hypertension, lacidipine

## Abstract

**Background:**

It has been shown that administration of lacidipine markedly reduces systolic blood pressure in elderly patients with hypertension without increasing the incidence of cardiovascular events and total mortality. But in Korea, there were no available data about the effectiveness and safety of lacidipine.

**Objectives:**

The goal of our study was to compare the effect of lacidipine and amlodipine besylate on sitting systolic blood pressure (SBP) and edema regression time as primary parameters, and sitting diastolic blood pressure (DBP) and tolerability as a secondary parameter in patients with hypertension.

**Method:**

This was a prospective, randomized, open-label, noninferiority study in which patients received 14 weeks of treatment with either lacidipine or amlodipine besylate. Patients aged 55 to 80 years having uncomplicated, mild-to-moderate essential hypertension (SBP 140 to <180 mm Hg or DBP ≥90 mm Hg) and receiving no antihypertensive medications during the 2 weeks before randomization were randomly assigned to receive lacidipine or amlodipine. The incidence of adverse events was also assessed.

**Results:**

In total, 315 patients (154 men, mean age 67.6 years) were included in the intent-to-treat analysis and randomly assigned to receive lacidipine (n = 162) or amlodipine besylate (n = 153); 286 patients were included in the per-protocol analysis (n = 150 for lacidipine, n = 136 for amlodipine) (12 in the lacidipine group and 17 in the amlodipine group were excluded from the per-protocol analysis due to consent withdrawal or protocol violation). There were no differences in demographic profiles between the 2 groups. Mean (SD) SBP changes at 14 weeks were −18.9 (12.7) mm Hg in the lacidipine group and −20.6 (12.4) mm Hg in the amlodipine group (*P* >0.05). Because the 1-sided 95% CI for the difference in mean SBP changes between groups (−4.18 to 0.72) was within the pre-specified lower limit (−5 mm Hg), lacidipine was considered noninferior to amlodipine. There were no differences in mean edema regression time and in mean DBP changes. These results were consistent in the isolated systolic hypertension subgroup analysis. The overall incidence of clinical adverse events was comparable between the 2 groups (ie, 7.4% in the lacidipine group and 11.1% in the amlodipine group [*P* >0.05]). The most common adverse events were headache and facial flushing (5 out of 162 patients [3.1%] in the lacidipine group and 11 out of 153 patients [7.2%] in the amlodipine group].

**Conclusions:**

Fourteen weeks of lacidipine treatment significantly reduced blood pressure in older Korean patients with mild-to-moderate hypertension. The efficacy of lacidipine was not inferior to that of amlodipine besylate and tolerability was comparable between the 2 treatment groups. ClinicalTrials.gov identifier: NCT00460915.

## Introduction

Arterial hypertension is a major public health problem in developed as well as developing countries. It has been shown that adequate control of blood pressure could reduce cardiovascular morbidity and mortality. Changing patterns of blood pressure occur with increasing age. In elderly patients, systolic blood pressure (SBP) increases progressively with age, whereas diastolic blood pressure (DBP) increases until age 55 or 60 years then tends to decrease later in life. Hypertension in elderly patients is characterized principally by increased systolic blood pressure, predominantly isolated systolic hypertension (ISH), in contrast to the patterns in young adults.^1^ ISH is considered a clinically very important cardiovascular risk factor because an increase in SBP alone, without an increase in DBP, can increase the incidence of cardiovascular and cerebrovascular events and all-cause mortality.^2,3^ However, several studies have shown that antihypertensive therapy also reduces cardiovascular morbidity and mortality in patients with ISH, indicating that this risk factor could be controlled with adequate antihypertensive treatment. The Systolic Hypertension in the Elderly Program^4^ study showed that treatment with thiazide reduced cardiovascular morbidity and mortality rates significantly more than those treated with placebo. In the Systolic Hypertension in Europe trial^5^ and Systolic Hypertension in China trial^6^ conducted in older patients with isolated SBP, similar results were observed after treatment with dihydropyridine calcium channel blockers (CCBs).

To date, many studies have been conducted to evaluate the effect of various antihypertensive drugs in elderly patients with hypertension; however, in many of these studies, only limited subgroup analyses of patients with either systolic or diastolic hypertension were performed,^7^ and no sufficient data on the prevention of hypertension-related complications in elderly patients were provided.

Lacidipine is a third-generation CCB in the dihydropyridine group. According to the The Systolic Hypertension in the Elderly: Lacidipine Long-term Study^8^ conducted in elderly patients with isolated systolic hypertension, lacidipine could reduce SBP effectively and safely; there was no significant difference in the cardiovascular morbidity and mortality between the lacidipine group and the chlorthalidone group. However, there is insufficient data on the effects of lacidipine on the older Korean population. The aim of our study was to compare the safety and efficacy of lacidipine with amlodipine besylate, the most commonly used calcium antagonist in Korea.

## Patients and Methods

This was a Phase IV, 14-week, prospective, randomized, open-label, noninferiority clinical trial conducted at 13 sites in Korea. Patients were enrolled and randomized from February 2007 to August 2009, and the randomized patients were followed until January 2010. The study protocol was approved by both national and regional review boards. This study was conducted in accordance with the Declaration of Helsinki/Good Clinical Practice Guideline and its amendments.

## Inclusion and Exclusion Criteria

This study was conducted in Korean adults aged between 55 and 80 years who had uncomplicated, mild-to-moderate essential hypertension (sitting SBP [SiSBP] ≥140 mm Hg or sitting DBP [SiDBP] ≥90 mm Hg, after a 2-week washout).

Patients were excluded from this study if they had severe hypertension (SiSBP ≥180 mm Hg), secondary hypertension, a history of myocardial infarction or unstable angina pectoris, congestive heart failure, anemia (hemoglobin <10.0 g/dL), renal disease (serum creatinine concentration >1.4 mg/dL), coagulopathy, peripheral arterial disease, hepatic disease (aspartate aminotransferase [AST] or alanine aminotransferase [ALT] >2.5-fold the upper limit of normal), uncontrolled diabetes (glycosylated hemoglobin [HbA_1c_] >8.0%), a history of metabolic acidosis or diabetic ketoacidosis, cancer, known hypersensitivity to CCBs, alcoholism, or other drug addiction.

Written informed consent was obtained from all eligible patients before the study.

## Treatments

Patients had study visits at the beginning and end of the 2-week washout period and at Week 2, 4, 6, 10, and 14 of drug treatment. Safety dropout evaluation was performed 2 weeks after the end of treatment (Week 16 of drug treatment) via telephone interview or clinic visit. Patients were randomly assigned, using a method of stratified block randomization by site, to receive either lacidipine or amlodipine besylate for 14 weeks.

For the first 2 weeks, patients received either lacidipine 2 mg or amlodipine 2.5 mg in the morning. Patients achieving the target blood pressure level (SBP <140 mm Hg and DBP <90 mm Hg) at Week 2 were maintained at the initial dose. Patients who did not achieve the target blood pressure level were titrated to lacidipine 4 mg or amlodipine 5 mg. At Week 6 of drug treatment, patients who failed to reach the target blood pressure level were titrated to lacidipine 6 mg or amlodipine 10 mg, and those who still failed to reach the target blood pressure level at Week 10 were given thiazide diuretics (ie, hydrochlorodichlozide 12.5 mg) in addition. Concomitant use of statins or thiazolidinediones was permitted during a 14-week treatment period; however, dose increase or reduction was not allowed. Other medications or substances that may interact with the study drug were prohibited during the study period. These include other types of antihypertensive medications, psychotropic agents, oral steroids, daily nonsteroidal anti-inflammatory drugs, estrogen, and antioxidant vitamin supplements.

## Assessments

### Effectiveness

At the initial screening visit, patients underwent a complete physical examination, medical history, laboratory assessment, baseline electrocardiography test, and chest x-ray. Laboratory analysis of collected blood samples was done at sites certified by the Korean Association of Quality Assurance for Clinical Laboratories and have regular follow-up of quality-care performance. Laboratory parameters included complete blood cell count, blood chemistry (ie, potassium, creatinine, calcium, AST, ALT, albumin, alkaline phosphatase, total bilirubin, prothrombin time, HbA_1c_, glucose, total cholesterol, HDL cholesterol, LDL cholesterol, triglyceride, high-sensitivity C-reactive protein [hs-CRP], and chest radiography. Laboratory analysis was verified according to prespecified normal ranges accepted by the institutional review board at the study center and a central coordinating committee.

At each clinic visit, sitting cuff SBP, DBP, heart rate, body weight, and height were recorded. To ensure consistency of through values, these measurements were taken at the same time of day. For each patient, blood pressure and heart rate were measured in each arm after patients were seated for at least 5 minutes, using a noninvasive oscillometric automatic blood pressure monitor (MX3; Omron Healthcare, Hoofddorp, the Netherlands). Three consecutive recordings were taken, each separated by at least 5 minutes. The mean of the 2 higher blood pressure values was calculated and recorded. Patients were excluded from the analysis if they had a discrepancy of >5 mm Hg between any 2 mean blood pressure measurements or a discrepancy of >20 mm Hg between the blood pressure measurements in both arms.

Edema regression time (ERT) was measured at the pretibial area twice during the study, at baseline (first visit) and 14 weeks after treatment.

The primary efficacy end points were the mean change in SiSBP from baseline to Week 14 and the mean change in ERT from baseline to Week 14. The secondary efficacy end points were the incidence of adverse events in each treatment group and the mean change in SiDBP from baseline to post-treatment Week 14.

### Tolerability

At each study visit, tolerability was assessed by evaluating medical history and laboratory measurements. Investigators collected information about laboratory measurements and clinical adverse events (AEs) by asking each patient a standardized question. All AEs occurring after treatment until the follow-up visit or the last visit were recorded in case report forms and reported appropriately. Furthermore, all AEs were evaluated by the investigator for relationship to the study drug (ie, definitely related, probably related, possibly related, probably not related, definitely not related, or unknown) and for strength of event (ie, mild, moderate, or severe). A clinical AE that results in death, is life-threatening, requires hospitalization or prolongation of existing hospitalization, results in significant permanent disability, or congenital anomaly was defined as a serious AE. If a laboratory or clinical AE was considered to be associated with the study drug and was serious enough to merit possible discontinuation of treatment, the clinical investigators determined if treatment with the study drug should be discontinued.

### Compliance evaluation

To determine the compliance rate, the investigator counted all unused medications returned by patients at each visit. Compliance rate was calculated as follows:

Compliance rate (%) = number of drugs actually taken / duration of prescription × 100.

Information about compliance to study treatment was recorded in the case report forms, including a reason for missed dose, if applicable. Patients who had compliance rate <70% at more than 2 visits were withdrawn from the study.

## Statistical Analysis

This is a noninferiority study. To demonstrate that the efficacy of lacidipine was not inferior to that of amlodipine, the lower bound of the 2-sided 90% (1-sided 95%) CI for the difference in SBP between the 2 groups after 14 weeks of treatment was calculated, and evaluated in comparison with the prespecified noninferiority margin of -5 mm Hg. If the lower bound of the 1-sided 95% CI is above -5 mm Hg, lacidipine would be assumed to be noninferior to amlodipine. The analyses of all other efficacy end points were performed at 2-sided significance level <5%.

The efficacy analysis population for this study was the intent-to-treat (ITT) population and per-protocol (PP) population. The ITT population consisted of all randomized patients. Of the ITT population, those who did not complete the 14 weeks of treatment had a compliance rate of <70% for 2 times or more, or had used a prohibited medication during the study were excluded from the PP population.

The efficacy analyses were performed on both ITT and PP populations; and the primary efficacy analysis was performed using the PP population analysis. For imputation of missing data for Week 14 SiSBP, ERT, or SiDBP, the last-observation-carried-forward method was used. The safety analysis was performed on the ITT population.

Baseline characteristics were compared between the 2 treatment groups. Continuous variables were analyzed using the Student *t* test, and categorical variables were analyzed and compared between 2 groups using χ^2^ test or Fisher’s exact test. Whether or not treatment effect was affected by baseline characteristics was analyzed using analysis of covariance. Incidences of AEs were analyzed and compared between the 2 groups, using χ^2^ test or Fisher’s exact test. Differences were considered statistically significant if *P* ≤ 0.05.

## Results

### Baseline characteristics

A total of 322 patients were enrolled in this study, and 317 of them provided written consent.

**Figure 1:**
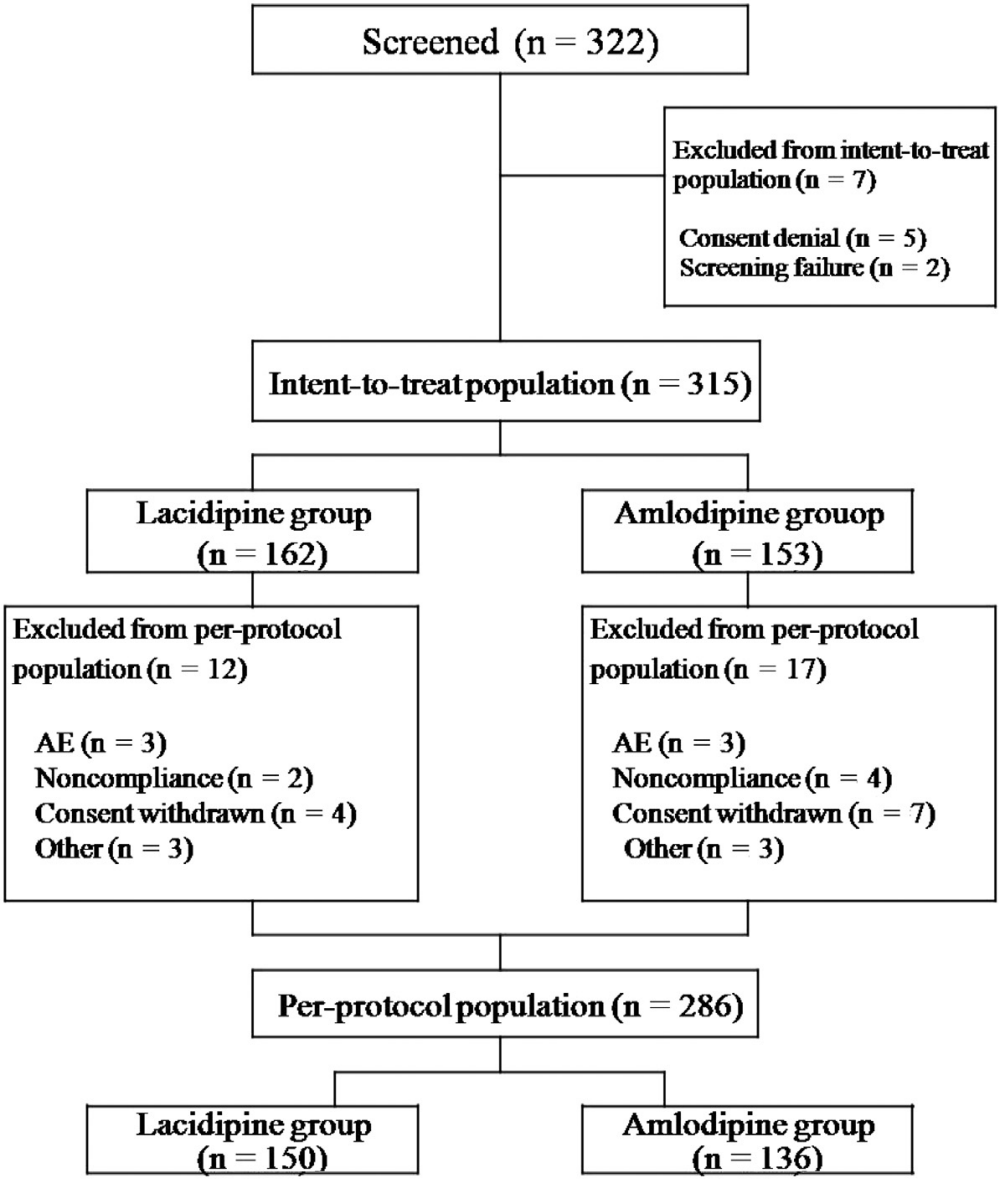
Flow diagram of study patients. AE, adverse event.

Excluding 2 patients who failed the screening, 315 patients were included in the ITT population (154 men, mean age 67.6 years, mean body weight 62.6 kg) and were randomly assigned to receive lacidipine (n = 162) or amlodipine besylate (n = 153) (Figure 1).

There was no significant difference in mean (SD) age between the lacidipine group (68.1 [6.1] years [range 56–83 years] and amlodipine group (67.1 [5.7] years [range 56–82 years]). There was also no significant difference between the 2 groups in sex (lacidipine 79 men [49%], amlodipine 75 men [49%]). Baseline SiSBP and SiDBP were comparable between the treatment groups, with 152.1 (10.3) mm Hg and 90.6 (8.9) mm Hg in the lacidipine group and 151.9 (10.9) mm Hg and 90.7 (9.5) mm Hg in the amlodipine group, respectively (Table I). With regard to baseline physical examination and medical history such as history of antihypertensive treatment, there was no significant difference between the 2 groups. Furthermore, there were no patients showing clinically significant abnormalities in 12-lead electrocardiography and chest x-ray. With regard to clinical laboratory values, there was no significant difference between the 2 groups in hemoglobin, electrolyte, AST, ALT, total bilirubin, prothrombin time, or HbA_1C_.

There was no significant difference in the dropout rate between the 2 groups. Of 315 patients randomized, 29 (9.2%) were early discontinued from the study: 12 patients from the lacidipine group and 17 patients from the amlodipine group. There were 3 patients (1.9%) (1 patient with each of skin rash, severe headache, and facial flushing) in the lacidipine group and 3 patients (1 patient with each of facial flushing, drowsiness, and severe fatigue) in the amlodipine group, who discontinued the study due to AEs. A total of 6 patients (1.9%) were discontinued due to noncompliance, and 11 patients (3.5%) were discontinued due to consent withdrawal. Thus PP population who completed the study according to the protocol contained 286 patients (n = 150 for lacidipine, n = 136 for amlodipine) (Figure 1).

## Effectiveness

In older Korean patients with mild-to-moderate hypertension, both PP and ITT analyses were performed to assess the primary end point and to compare the therapeutic effect of lacidipine and amlodipine.

The difference in change in mean SiSBP between the lacidipine and amlodipine groups was 1.7 mm Hg (95% CI -3.4 to 1.3). Because the 95% CI for the difference between groups was within the lower limit of -5 mm Hg, lacidipine was considered noninferior to amlodipine (Table II).

**Figure 2:**
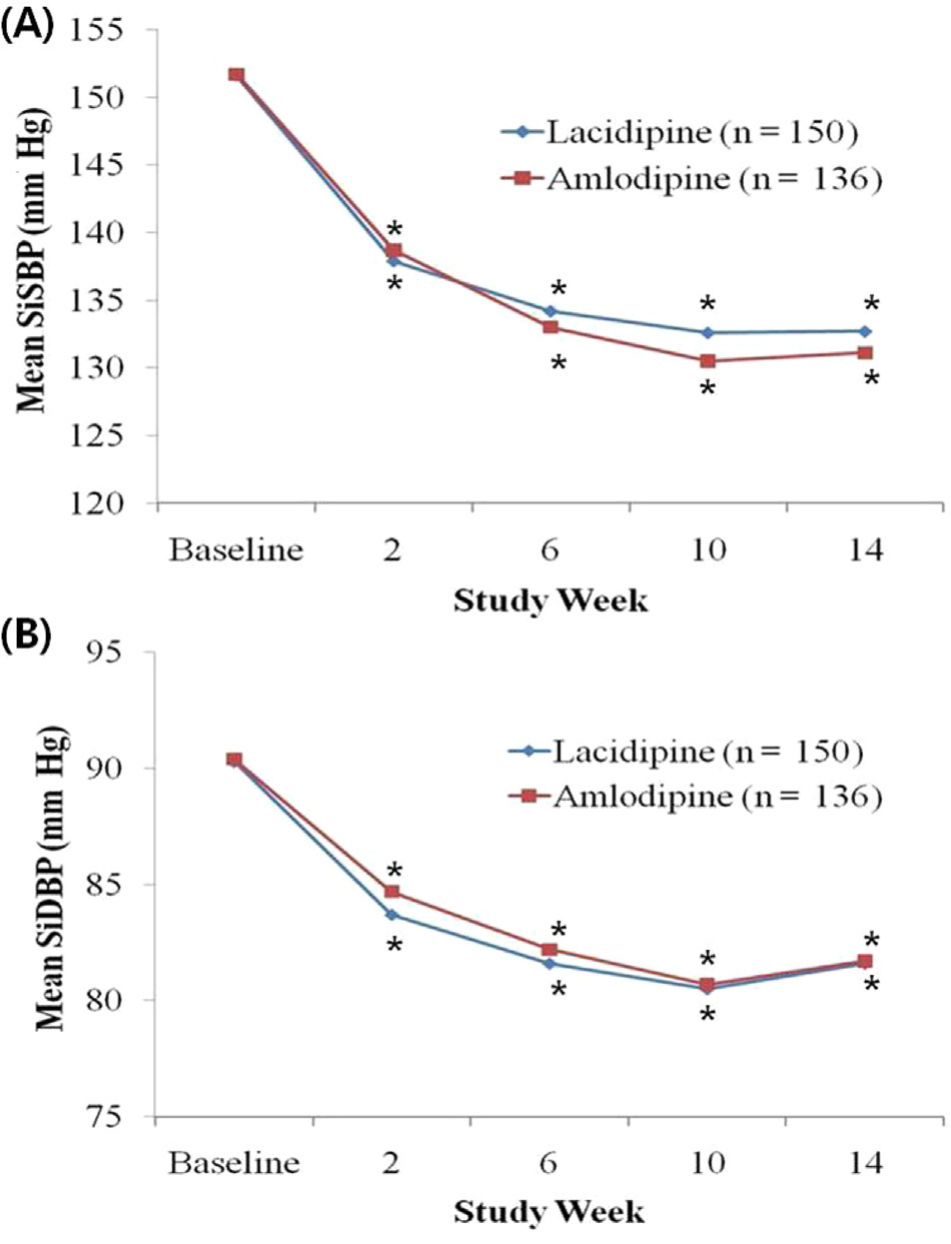
Sitting (A) systolic blood pressure (SiSB) and (B) diastolic blood pressure (SiDBP) changes during 14-week treatment with lacidipine or amlodipine in older Korean patients with mild-to-moderate hypertension (per-protocol population). ^*^P<0.001 versus baseline. No significant differences were found between the 2 groups.

In the PP analysis, mean SiSBP decreased significantly from baseline after 14 weeks of treatment in the 2 groups (lacidipine from 151.6 [10.2] mm Hg to 132.7 [11.1] mm Hg, amlodipine from 151.7 [11.2] mm Hg to 131.1 [11.2] mm Hg; both *P* < 0.001) (Figure 2). The difference between the 2 groups in mean change in SiSBP from baseline to Week 14 was not significant (lacidipine -18.9 [12.7] mm Hg, amlodipine, -20.6 [12.4] mm Hg; *P* = 0.25) (Table II).

Mean SiDBP decreased significantly after 14 weeks of treatment in the 2 groups (lacidipine from 90.3 [9.0] mm Hg to 81.6 [7.4] mm Hg, amlodipine from 90.4 [9.7] mm Hg to 81.7 [7.1] mm Hg; both *P* < 0.001) (Figure 2). The difference between the 2 groups in mean (SD) change in SiDBP from baseline to Week 14 was not significant (lacidipine -8.7 [8.3] mm Hg, amlodipine, -8.7 [8.6] mm Hg; *P* = 0.95) (Table II).

**Figure 3:**
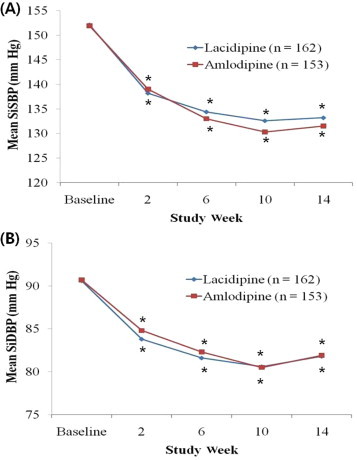
Sitting (A) systolic blood pressure (SiSBP) and (B) diastolic blood pressure (SiDBP) changes during 14-week treatment with lacidipine or amlodipine in older Korean patients with mild-to-moderate hypertension (intent-to-treatment population). ^*^P < 0.001 versus baseline. No significant differences were found between the 2 groups.

In the ITT analysis, mean (SD) SiSBP decreased significantly from baseline after 14 Weeks of treatment in the 2 groups (lacidipine from 152.1 [10.3] mm Hg to 133.2 [11.3] mm Hg, amlodipine from 151.9 [10.9] mm Hg to 131.5 [11.3] mm Hg; both *P* < 0.001) (Figure 3). The difference between the 2 groups in mean (SD) change in SiSBP from baseline to Week 14 was not significant (lacidipine -18.4 [12.7] mm Hg, amlodipine -19.4 [12.7] mm Hg; *P* = 0.47) (Table II).

Mean (SD) SiDBP decreased significantly after 14 weeks of treatment in the 2 groups (lacidipine from 90.6 [8.9] mm Hg to 81.8 [7.4] mm Hg, amlodipine, from 90.7 [9.5] mm Hg to 81.9 [7.2] mm Hg; both *P* < 0.001) (Figure 3). The difference between the 2 groups in mean (SD) change in SiDBP from baseline to Week 14 was not significant (lacidipine -8.6 [8.3] mm Hg, amlodipine -8.3 [8.5] mm Hg; *P* = 0.74) (Table II).

The difference between the 2 groups in SiSBP response rate did not reach statistical significance, with 73.5% of patients (119 out of 162) in the lacidipine group and 78.4% (120 out of 153) of those in the amlodipine group achieving this end point. The difference between the 2 groups in SiDBP response rate also was not significant, with 81.5% of patients (132 out of 162) in the lacidipine group and 83.7% of patients (128 out of 153) in the amlodipine group achieving this end point (Table III).

The result of SiSBP and SiDBP changes in the both ITT analysis and PP analysis had similar patterns in both groups (all *P* < 0.001 vs baseline) (Figures 2 and 3).

**Figure 4:**
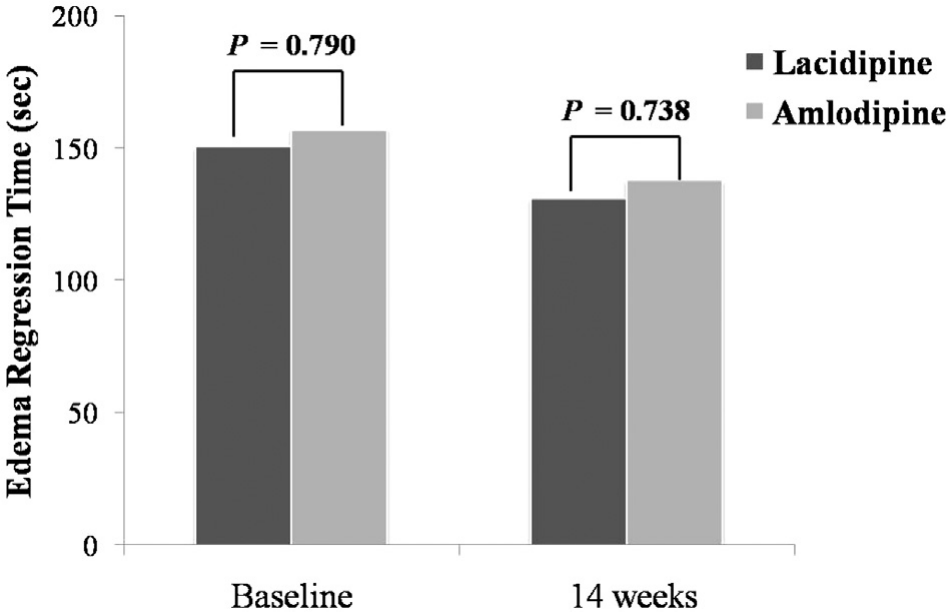
Comparison of edema regression time between lacidipine and amlodipine groups at baseline and 14 weeks after treatment. No significant between-group differences were found.

The changes in ERT from baseline to Week 14 are presented in the Figure 4. ERT measured at baseline and at the end of Week 14 showed no statistically significant difference between the lacidipine group and amlodipine group at both time points (all *P* > 0.05).

## Subgroup Analysis of patients with ISH

The number of patients who had ISH (SiSBP ≥ 140 mm Hg and SiDBP <90 mm Hg) was 60 in the lacidipine group and 58 in the amlodipine group (PP analysis). No significant difference between the 2 groups was observed in baseline clinical and laboratory characteristics. There was no significant difference between the 2 groups in mean (SD) age (lacidipine, 69.1 [6.3] years, amlodipine, 68.1 [5.21] years; *P* = 0.36), SiSBP (lacidipine 148.8 [8.0] mm Hg, amlodipine 147.3 [6.9] mm Hg; *P* = 0.26), SiDBP (lacidipine 82.1 [5.9] mm Hg, amlodipine 81.4 [7.1] mm Hg; *P* = 0.67), ERT (lacidipine 198.4 [216.7] seconds, amlodipine 224.1 [240.2] seconds; *P* = 0.75), and hs-CRP (lacidipine 2.52 [4.47] mg/L, amlodipine 1.10 [1.47] mg/L; *P* = 0.33).

**Figure 5:**
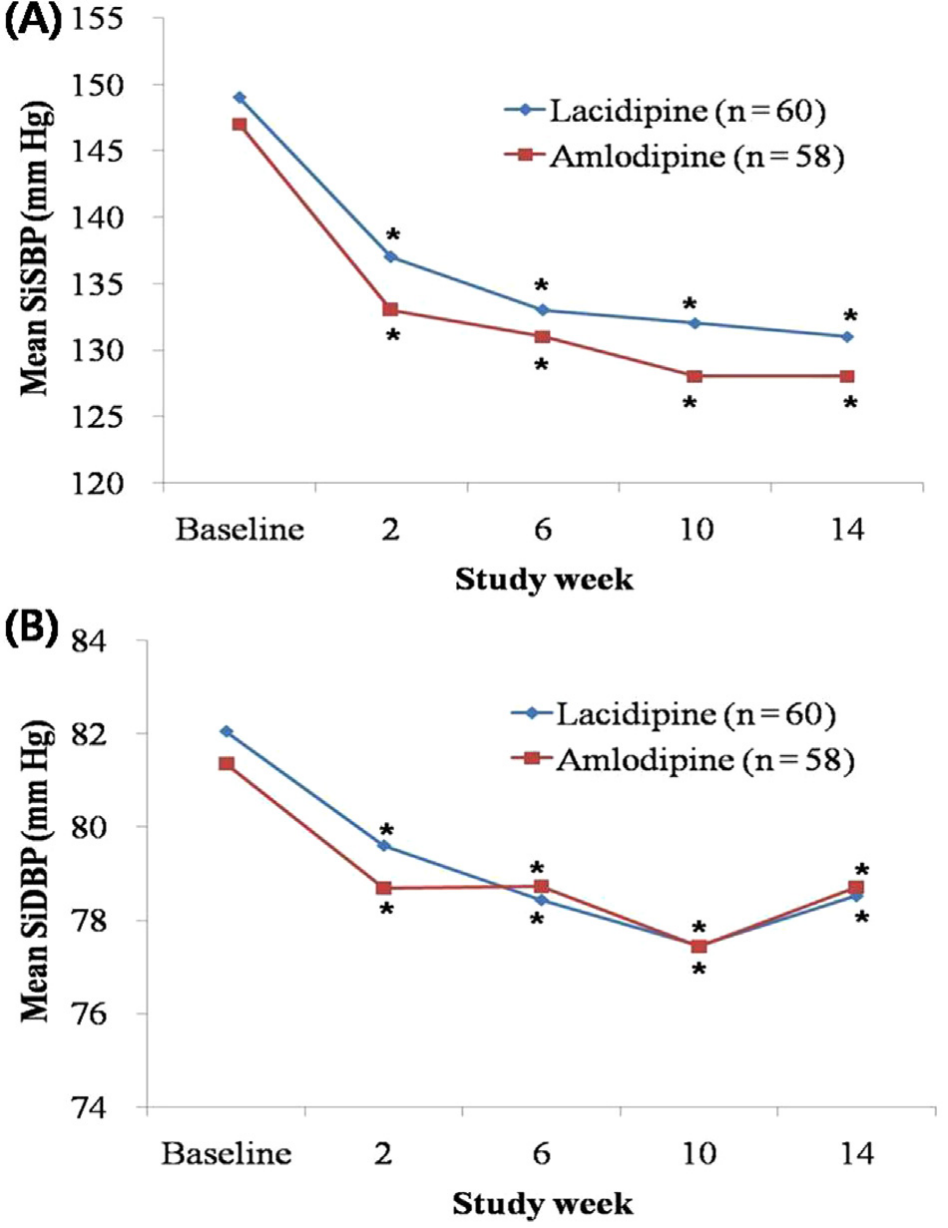
Subgroup analysis of sitting (A) systolic blood pressure (SiSBP) and (B) diastolic blood pressure (SiDBP) changes during 14-week treatment with lacidipine or amlodipine in older Korean patients with isolated systolic hypertension (per-protocol population). ^*^P < 0.001 versus baseline. No significant differences were found between two groups.

There was no significant difference between the 2 groups in the blood pressure-lowering effect from baseline to Week 14 (Figure 5). There was no significant difference between the 2 groups in mean (SD) change in SiSBP (lacidipine -18.1 [11.9] mm Hg; amlodipine -19.7 [12.3] mm Hg; *P* = 0.62), SiDBP (lacidipine -3.5 [7.0] mm Hg; amlodipine -2.7 [7.7] mm Hg; *P* = 0.52), and ERT (lacidipine -23.4 [63.8] seconds; amlodipine -34.5 [112.2] seconds; *P* = 0.52).

## Tolerability

There was no significant difference between the 2 groups in the rate of clinical and laboratory AEs (Table IV). The rate of overall AEs was 7.4% (12 patients in 162 patients) in the lacidipine group and 11.1% (17 patients in 153 patients) in the amlodipine group (*P* > 0.05).

The most common drug-related AEs were headache (n = 3), facial flushing (n = 2), and dizziness (n = 2) in the lacidipine group, and headache (n = 6), facial flushing (n = 5) in the amlodipine group. In addition, 1 patient in the lacidipine group and 5 patients in the amlodipine group reported low extremity edema. Cardiovascular system, general, and skin AEs were comparable between the 2 groups, and no significant difference between the 2 groups in the rate of other AEs was observed.

Laboratory analysis, including blood cell count and blood chemistry, showed no abnormal finding in either group, as verified by prespecified normal ranges accepted by the institutional review board at each study center and the central coordinating committee. The number of patients who required treatment discontinuation due to drug-related clinical AEs determined by investigators was 3 in the lacidipine group (1 patient with each of skin rash, severe headache, and facial flushing) and 3 in the amlodipine group (1 patient with each of facial flushing, drowsiness, and severe fatigue).

## Discussion

According to a MEDLINE search using the terms *lacidipine* and *amlodipine besylate*, this was the first prospective, randomized, head-to-head comparison of lacidipine and amlodipine in the older patients with hypertension. In patients with hypertension aged 55 years or older, the effectiveness and AEs of 2 CCBs, lacidipine and amlodipine, were compared. Overall, in this 14-week, multicenter, prospective, randomized, open-label, noninferiority clinical trial, lacidipine was found to be noninferior to amlodipine besylate in the reduction of SiSBP and SiDBP, SiSBP reduction rate, clinical and laboratory AE rate, and overall tolerability profiles in older Korean population with mild-to-moderate hypertension both in the PP and ITT analysis.

SBP is an independent risk factor for stroke, coronary artery disease, heart failure, and renal failure.^9^ SBP tends to increase linearly with age, whereas DBP increases in parallel with SBP until age of 55 years, and then tends to decrease after age of 55 years due to increased central arterial stiffness.^1^ Therefore, the prevalence of ISH increases with age, and ISH is considered an important cardiovascular or cerebrovascular risk factor. In addition, the hemodynamic pattern is different between young and older patients with hypertension. Most young patients with hypertension tend to maintain the hyperkinetic state, with increased heart rate and consequently increased cardiac index and an increase in left ventricular ejection fraction. In contrast, older patients with hypertension tend to have decreased systolic function and subsequently depressed cardiac reserve, which results in impaired diastolic function at rest and markedly decreased diastolic function during exercise, compared with young patients with hypertension. Thus, it is very important to attain adequate blood pressure control in older patients. With adequate control of blood pressure, cardiovascular morbidity and mortality can be significantly reduced in elderly patients with hypertension.^4,5,10,11^

Antihypertensive medications that are effective in elderly persons include CCBs and diuretics. Amlodipine is widely used for the treatment of hypertension and angina pectoris and has a relatively fast onset of activity. The effectiveness of amlodipine has been demonstrated in many studies. The Valsartan Antihypertensive Long-Term Use Evaluation study^12^ conducted in 15,245 patients with hypertension showed that patients treated with amlodipine 5 to 10 mg/d had a more significant reduction in SBP and DBP than those treated with valsartan 150 to 300 mg/d (*P*<0.001).^12^ In the Comparison of Amlodipine vs Enalapril to Limit Occurrences of Thrombosis trial,^13^ patients with coronary artery disease and normal blood pressure who were treated with amlodipine 10 mg/d over 2 years had significantly lower rates of cardiovascular events than those in the placebo group (*P* = 0.03); however, the study failed to demonstrate that the use of amlodipine might prevent progression of atherosclerosis, compared with placebo.

In contrast, lacidipine, a third-generation lipophilic dihydropyridine CCB, has intrinsically slow onset of activity, which results in less reflex tachycardia^14^ and long duration of action and high degree of vascular selectivity. The results of this study demonstrated that therapeutic effectiveness of lacidipine in reducing SBP and DBP was comparable to that of amlodipine, and we believe these findings imply comparable cardiovascular event rate reduction. Findings in the European Lacidipine Study on Atherosclerosis,^15^ in which 2334 patients aged 45 to 75 years who had mild-to-moderate essential hypertension were followed, showed that the lacidipine group had significantly lower atherosclerotic progression and plaque formation in the carotid artery than the atenolol group. Because the extent of blood pressure reduction of Korean patients in this study was comparable to that of European patients, long-term follow-up for cerebrovascular event was deemed necessary; however, this study could not provide data on cardiovascular event rate due to the limited length of follow-up. Thus, in an Asian country where the prevalence of cerebrovascular events is relatively high, further evaluation is clearly warranted.

According to the subgroup analysis in patients with ISH, the reduction in SBP in the subgroup was consistent with that in the total population, but the reduction in DBP in the subgroup was relatively lower. Thus, it was concluded that in patients with ISH who have normal DBP, lacidipine is as effective and safe as amlodipine.

There was also no significant difference between the lacidipine group and amlodipine group in ERT, indicating that the rate of edema, a most common AE associated with CCBs, in the lacidipine group was comparable to that in the amlodipine group.

The rates of AEs, including those of the autonomic nervous system and cardiovascular system, dermatologic, and laboratory, were not significantly different between the 2 groups. In previous studies,^16^ the rate of edema related to the use of CCBs was reported up to 34%; however, the occurrence rate of low extremity edema (lacidipine 0.6%, amlodipine 3.3%) and facial flushing (lacidipine 1.2%, amlodipine 3.3%) was very low in this study, and there was no significant difference between the 2 groups in ERT. Three patients from each of the 2 groups were discontinued from the study due to AEs, but none experienced a serious AE that required hospitalization or was life-threatening. Furthermore, the drug compliance rate was comparable between the 2 groups. There was no significant difference in the rate of early discontinuation from the study between the 2 groups.

Limitations of this study include a relatively small sample size, short duration of follow-up, and use of clinical blood pressure instead of 24-hour blood pressure monitoring.

## Conclusions

In this prospective study, we found that lacidipine was noninferior to amlodipine besylate in the reduction of blood pressure in older Korean patients with uncomplicated, mild-to-moderate essential hypertension or ISH. The safety and tolerability of lacidipine were consistent with those of amlodipine besylate.

## Conflicts of Interest

The authors have indicated that they have no conflicts of interest regarding the content of this article.

## Figures and Tables

**Table I:** Baseline characteristics of study patients.

Characteristic	Lacidipine (n = 162)	Amlodipine (n = 153)	*P*
Age, y			0.16
Mean (SD)	68.1 (6.1)	67.1 (5.7)	
Range	56–83	56–82	
Sex, n (%)			0.96
Men	79 (48.8)	75 (49.0)	
Women	83 (51.2)	78 (51.0)	
Height, cm			0.74
Mean (SD)	159.5 (9.1)	159.9 (9.3)	
Range	137–184	140–188	
Weight, cm			0.70
Mean (SD)	62.3 (8.9)	62.7 (10.9)	
Range	42–91	37–100	
Smoking, n (%)			0.08
Current smoker	13 (8.0)	22 (14.4)	
Exsmoker	44 (27.2)	29 (19.0)	
Nonsmoker	105 (64.8)	102 (66.7)	
Alcohol use, n (%)			0.74
Yes	58 (35.8)	52 (34.0)	
No	104 (64.2)	101 (66.0)	
Blood pressure, mm Hg			
Sitting systolic			0.85
Mean (SD)	152.1 (10.3)	151.9 (10.9)	
Range	131–180	131–180	
Sitting diastolic			0.90
Mean (SD)	90.6 (8.9)	90.7 (9.5)	
Range	62–120	54–109	
Heart rate, bpm			0.35
Mean (SD)	72.2 (9.5)	71.2 (9.1)	
Range	52–98	50–102	
Glycosylated hemoglobin, %			0.68
Mean (SD)	5.9 (0.8)	5.9 (0.7)	
Range	3.5–8.0	4.2–8.0	

**Table II:** Blood pressure parameters at baseline and end of treatment (Week 14) with lacidipine or amlodipine in an elderly Korean population.

Parameter	Lacidipine	Amlodipine	*P*
	←----------Mean (SD)----------→		
Per-protocol anaylsis			
n	150	136	
SiSBP, mm Hg			
Baseline	151.6 (10.2)	151.7 (11.2)	0.94
Week 14	132.7 (11.1)*	131.1 (11.2)*	0.22
Change	−18.9 (12.7)	−20.6 (12.4)	0.25
SiDBP, mm Hg			
Baseline	90.3 (9.0)	90.4 (9.7)	0.96
Week 14	81.6 (7.4)*	81.7 (7.1)*	0.89
Change	−8.7 (8.3)	−8.7 (8.6)	0.95
Intent-to-treat analysis			
n	162	153	
SiSBP, mm Hg			
Baseline	152.1 (10.3)	151.9 (10.9)	0.85
Week 14	133.2 (11.3)*	131.5 (11.3)*	0.19
Change	−18.4 (12.7)	−19.4 (12.7)	0.47
SiDBP, mm Hg			
Baseline	90.6 (8.9)	90.7 (9.5)	0.90
Week 14	81.8 (7.4)*	81.9 (7.2)*	0.88
Change	−8.6 (8.3)	−8.3 (8.5)	0.74

SiDBP, sitting diastolic blood pressure; SiSBP, sitting systolic blood pressure.

⁎ *P* < 0.001 versus baseline.

**Table III:** Response rates with 14-week treatment with lacidipine or amlodipine in elderly Korean patients (intent-to-treat population).*

Parameter	Lacidipine (n = 162)	Amlodipine (n = 153)
SiSBP†	119 (73.5)	120 (78.4)
SiDBP‡	132 (81.5)	128 (83.7)

SiDBP, sitting diastolic blood pressure; SiSBP, sitting systolic blood pressure.

⁎ Values are presented as n (%). No significant between-group differences were found.

† Response defined as <140 mm Hg at Week 14.

‡ Response defined as <90 mm Hg at Week 14 or an absolute reduction of >10 mm Hg.

**Table IV:** Adverse events (AEs) during 14-week treatment with lacidipine or amlodipine in elderly Korean patients with uncomplicated, mild-to-moderate, essential hypertension (intent-to-treat population).*

AE	Lacidipine (n = 162)	Amlodipine (n = 153)
Nervous system		
Headache	3 (1.9)	6 (3.9)
Dizziness	2 (1.2)	1 (0.7)
Drowsiness	0	1 (0.7)
Hoarseness	0	0
Tremor	0	0
Cardiovascular system		
Chest discomfort	0	0
Dyspnea	1 (0.6)	0
Palpitation	1 (0.6)	1 (0.7)
Syncope	0	0
General		
Fatigue	0	1 (0.7)
Facial flushing	2 (1.2)	5 (3.3)
Edema, low extremity	1 (0.6)	5 (3.3)
Dry mouth	0	0
Skin		
Rash	1 (0.6)	0
Laboratory		
AST/ALT elevation	0	1 (0.7)
Dyslipidemia	1 (0.6)	2 (1.3)
Anemia	0	0
Total patients		
With at least 1 AE	12 (7.4)	17 (11.1)
With no AEs	150 (92.6)	136 (88.9)
Withdrawn due to AEs	3 (1.9)	3 (2.0)

ALT, alanine aminotrasferase; AST, aspartate aminotransferase.

⁎ Values are presented as n (%). Some patients experienced >1 AE. No significant between-group differences were found.
